# Shared decision-making in general practice from a patient perspective. A cross-sectional survey

**DOI:** 10.1080/02813432.2022.2069700

**Published:** 2022-04-28

**Authors:** Birgitte Nørgaard, Signe Beck Titlestad, Michael Marcussen

**Affiliations:** a Department of Public Health, University of Southern Denmark, Odense C, Denmark; b Department of Clinical Research (OPEN), University of Southern Denmark, Odense C, Denmark

**Keywords:** General practice, shared decision-making, patient involvement, patient perspectives

## Abstract

**Objectives:**

We aimed to assess patient involvement in terms of shared decision-making in general practice from the perspectives of patients with chronic obstructive pulmonary disease (COPD) or type 2 diabetes (T2DM) (or both).

**Design:**

A cross-sectional survey using the 9-item Shared Decision-Making Questionnaire (SDM-Q-9) ranging from 0 to 5 (best).

**Setting and subjects:**

Patients diagnosed with either T2DM and/or COPD were asked to focus on their most recent consultation in general practice concerning their T2DM or lung disease. Responders were approached through the Danish Diabetes Association and Danish Lung Association.

**Results:**

The sample included 468 responders. Mean scores for the total sample were between 3.3 and 4.2. The overall mean score for all items was 3.7. The highest overall mean score was for patients with T2DM, whereas the lowest overall mean score was for patients having both T2DM and COPD. Furthermore, we observed a slightly lower overall mean score for women compared to men and for those younger than 65 years compared to those aged 65 years or older.

**Conclusion:**

Overall, patients are involved in shared decision-making in general practice Minor nuances were found because patients with COPD were less involved in shared decision-making compared to patients with T2DM. Similarly, younger patients and women were less involved than older patients and men.

## 1. Background

Patients should be involved in their care and treatment, as well as decisions concerning their lives. Shared decision-making is considered a means to actively involve patients in treatment and care [1]. Research has shown improved health outcomes when patients with chronic diseases are involved in the management of their own health condition and when their individual needs are considered [2,3]; this approach also reduces patients’ healthcare costs [3–6]. Many associated positive effects of patient involvement, including increased satisfaction with care and treatment [7], improved quality of life, enhanced mental health [8,9] and increased compliance [10], are well documented in research.

Involving patients in decisions regarding treatment and healthcare delivery further benefits healthcare professionals by enhancing their understanding of patients’ health problems and enabling them to deliver individualized and tailored healthcare [11]. Chronic obstructive lung disease (COPD) and type 2 diabetes (T2DM) are incurable diseases representing major health problems [12] and are primarily managed by general practitioners (GPs), [13–15]. Patients with COPD and/or T2DM tend to have complex pathways characterized by multiple contacts across the healthcare system [16]; thus, they need to cope with and manage their disease. Patient involvement is increasingly recognized as beneficial to chronic disease management [3], and knowledge of patients’ perspectives regarding their involvement in treatment is important to provide the best possible care.

This study aimed to assess patient involvement in terms of shared decision-making in general practice from the perspectives of patients with COPD or T2DM (or both).

## 2. Methods

The findings are reported according to the STROBE checklist for observational studies [17].

### 2.1. Study design

We performed a cross-sectional survey of patients diagnosed with either T2DM and/or COPD. Data were collected using the 9-item Shared Decision-Making Questionnaire (SDM-Q-9).

### 2.2. Setting and participants

Data were collected prospectively during March and April 2021 using web-based software (SurveyXact.dk). Responders were approached through the Danish Diabetes Association and Danish Lung Association. Both organizations provided contact to their members through their local branches. A unique link provided access to the questionnaire. To increase the response rate, a reminder was e-mailed twice, with a two-week interval. One week after the last reminder, the survey was closed. We included patients aged 18 years or older with COPD or T2DM (or both).

### 2.3. Variables and data source

The 9-item Shared Decision Making Questionnaire was developed in a theory-driven manner [18] and measures the extent to which patients are involved in the process of decision-making, patient version (SDM-Q-9), (a physician version SDM-Q-Doc is also available) [19,20]. The first version was developed in 2006 [18], but due to psychometric challenges, including the ceiling effect, the instrument has been revised [19]. The revised questionnaire has been translated into multiple languages, including Danish, and validated in a Danish setting showing a unidimensional factor with high factor loadings of more than 0.7 [21]. The instrument was developed for use in research and clinical practice and can be implemented for the purposes of evaluation and quality improvement in healthcare [19] but should, however, only be applied in the case of preference-sensitive decisions (i.e. when there are several treatment options for a particular disease) [20,21]. As recommended by the instrument developer, the responders were informed that they should focus on their most recent consultation in general practice concerning their T2DM or lung disease.

The SDM-Q-9 includes nine items concerning shared decision-making, all of them answered on a six-point Likert scale ranging from ‘Completely agree’ to ‘Completely disagree (three positive answers and three negative answers). At the end of the questionnaire, an open-ended question encourages the responders to add further comments. Prior to these items, we added items on informed consent (Agree/Do not agree), diagnosis (T2DM or lung disease, both or none), sex (male, female, other), age (continuous variable), and after the SDM-Q-9 items, we added information on how the responders could assess the final results afterwards, if interested.

### 2.4. Bias

We acknowledge that approaching responders through patients’ associations might imply biases, including self-selection and—perhaps more importantly—a risk that members of patients’ associations might represent increased socio-economic status compared to non-members [22]. This was, however, our best possibility to approach a large group of patients with similar diagnoses and with presumably frequent GP consultations. Furthermore, the COVID-19 outbreak prohibited in-person information, questions and answers and encouragement to increase both rigorousness and response rates.

### 2.5. Study size

Being a descriptive study, sample size calculations were not performed. We did, however, apply a threshold of at least ten responders per item [23] (i.e. at least 90 responders); in case this number was not reached, the analyses would not be carried out.

### 2.6. Quantitative variables and statistical methods

We included only completed questionnaires in the analyses (i.e. questionnaires with any missing data were omitted). If responders stated that they had been diagnosed with neither T2DM nor lung disease, their questionnaire was closed.

Reviewing the literature, heterogeneous methods regarding both score transformation and statistical analyses have been applied to analyze SDM-Q9 results [19,24,25]. However, we chose to follow the procedures applied in the validation study of the Danish version [21]. Thus, we assumed equally spaced categories, despite the ordinal nature of the response categories and answers being coded numerically to calculate means for each item and for the scale (Completely agree = 5, Completely disagree = 0), and we interpreted the mean score as the average assessment of perceived shared decision-making in a consultation. Furthermore, to explore associations, the response categories were grouped into either positive (the three positive answers) or negative (the three negative answers). Sex was treated as a bivariate variable. For the analysis, age was dichotomized into <65 and ≥65 years, agreeing with the official Danish threshold for geriatrics [26]. Data were analyzed by chi-square and Wilcoxon rank-sum testing using Stata, version 16 (StataCorp. 2021. Stata Statistical Software: Release 16. College Station, TX: StataCorp LP).

### 2.7. Ethical considerations

The study was approved by the Research Ethics Committee of the University of Southern Denmark (id 21/8757) in accordance with both research ethics and General Data Protection Regulation (GDPR) legislation [27]. The questionnaire was proceeded by information about the purpose of our study, contact information, anonymity, confidentiality and the responders’ right to withdraw from the study at any time. An informed consent box was ticked initially by all responders. According to Danish legislation, no further approval was required.

## 3. Results

### 3.1. Population and demographics

The sample included 627 responders. After removing incomplete questionnaires (*n* = 139) and responders stating that they had neither COPD nor T2DM (*n* = 30), we had a sample of 468 responders. Men numbered 45.6% and women 54.4%; their mean age was 68 years, SD 8.8 (68.9 years for men, SD 8.5 and 67.3 years for women, SD 9). For further details, see Table 1.

**Table 1.** Patient demographics by diagnosis.

	T2DM	COPD	T2DM & COPD	Total
	(*n* = 275)	(*n* = 143)	(*n* = 40)	(*n* = 458)
Sex				
Men	49.8 (137)	35.7 (51)	52.5 (21)	45.6 (209)
Women	50.2 (138)	64.3 (92)	47.5 (19)	54.4 (249)
Age, mean (SD)	66.3 (9.5)	70.6 (6.9)	71.1 (7.0)	68.0 (8.8)
Men	66.8 (8.8)	73.3 (6.2)	72.9 (5.3)	68.9 (8.5)
Women	65.8 (10.2)	69.1 (6.8)	69.3 (8.3)	67.3 (9.1)

Of the included responses, 29.9% stated that they had been diagnosed with COPD and 57.5% with T2DM, whereas 8.45% confirmed they had both.

### 3.2. Shared decision-making

Calculating item means revealed mean scores for the total sample between 3.3 (item 7) and 4.2 (item 1). The overall mean score for all items was 3.7. The highest overall mean score was found for patients with T2DM, whereas having both T2DM and COPD resulted in the lowest overall mean score. However, patients with COPD also presented an overall mean score lower than the total overall mean score. We also found a slightly lower overall mean score for women compared to men and for those younger than 65 years compared to those aged 65 years or older. Further details are presented in Table 2.

**Table 2.** Item mean scores per diagnosis (range 0–5).

Item^a^	Total	T2DM	COPD	T2DM & COPD	Men	Women	<65 yrs	65+ yrs
1. A decision needs to be made	4.2	4.3	3.9	4.3	4.3	4.1	4	4.3
2. How I want to be involved	3.8	3.8	3.7	3.7	3.9	3.7	3.6	3.8
3. Informing me that different options are available	3.9	3.9	3.8	3.7	3.9	3.8	3.7	3.9
4. Explaining to me the advantages and disadvantages	3.6	3.7	3.5	3.6	3.7	3.5	3.5	3.6
5. Helping me to understand the information	3.8	3.9	3.7	3.5	3.9	3.8	3.7	3.8
6. Asking about the treatment option I prefer	3.4	3.5	3.3	3.2	3.5	3.3	3.3	3.5
7. Weighing jointly the different options	3.3	3.4	3.2	3.1	3.5	3.2	3.2	3.4
8. Selecting a treatment option together	3.7	3.8	3.6	3.2	3.8	3.6	3.5	3.7
9. Agreeing on how to proceed	4.0	4.1	3.8	3.6	4.1	3.9	3.9	4
Overall mean score	3.7	3.8	3.6	3.5	3.8	3.7	3.6	3.8

^a^Item wording is truncated.

Regarding the differences between patients with T2DM and COPD, the chi-square analyses showed *tendencies* towards patients with COPD being less involved in shared decision-making than patients with COPD for all items, as also indicated by the mean scores. However, some significant differences were found, including Items 1 (*p*=.015), 5 (*p*=.02), 6 (*p*=.04), 8 (*p*=.007) and 9 (*p*=.004).

Regarding the tendency towards patients younger than 65 years being less involved in shared decision-making compared to patients aged 65 years or older, only Items 1 (*p*=.04) and 3 (*p*=.03) showed significant results.

Finally, regarding sex, we found women to be significantly less involved in shared decision-making only in Item 7 (*p*=.024).

Because psychometric tests of the SDM-Q9 questionnaire have shown tendencies towards the ceiling effect [21], this effect was also calculated in our study. Considering the ceiling effect when 15% or more answer the highest (5) item score [28], we found this for Items 1, 2, 3, 5, 8 and 9. The floor effect (15% or more score the lowest (0) item score) was found in Items 6, 7 and 8.

## 4. Discussion AND conclusion

### 4.1. Main findings

Overall, we found that both patients with T2DM and patients with COPD reported relatively high levels of shared decision-making in general practice with mean scores between 3.3 and 4.2 (range 0–5). However, the analyses revealed some minor differences. Firstly, patients with COPD reported lower mean scores of the SDM-Q9 (in all items and significantly in five of nine items) than patients with T2DM. Secondly, respondents younger than 65 years of age reported lower mean scores generally than respondents aged 65 years or older (significantly in two of nine items). Finally, women reported lower mean scores than men (significantly in one of nine items).

Even though responders with T2DM generally were younger than responders with COPD (mean 66.3 and 70.6, respectively) and, thus, should report as being less involved in shared decision-making, the overrepresentation of women among responders with COPD (64.3%) might contribute to the fact that responders with COPD overall tend to be less involved in shared decision-making than responders with T2DM.

### 4.2. Interpretation

Patients with COPD reported significantly lower mean scores than patients with TD2 in items 1, 5, 6 8 and 9 and those could raise the question whether these five items differ in their focus or relevance from the remaining four items. A study investigating what matters to patients with COPD found that range of issues mattered to people living with COPD, including meaning, purpose and relationships, ease of access to health services and the importance of being treated as a person [29]. Whether this corroborates the five SDM-Q9 items mentioned cannot be answered by our data. However, the fact that patients with COPD report to be less involved in their encounters with healthcare has also been found by other researchers. Schroedl *et al.* found that patients with COPD often suffer from emotional distress, depressive symptoms and anxiety due to their illness. Only half of these patients understand their disease severity and prognosis [30], and it appears that both patients and GPs ask for specialist support to make the right decisions about treatment and the future [30,31]. Moreover, patients with COPD experience stigma and blame, as well as from their healthcare providers [32], and a study has found that GPs are challenged in the encounter with patients with COPD. The GPs find it difficult to balance the patients’ perspectives against their professional estimation of the patients’ needs for treatment, and they strive to include the patients’ knowledge of their own body and illness in decision-making [33].

The fact that this survey was conducted during COVID-19 lock-down should be mentioned. The situation during COVID-19 has meant that GP consultations for not acutely ill patients have been either postponed, cancelled or changed into online sessions. This new approach is not necessarily without challenges because our respondent group is relatively old (mean age 68 years, SD 8.8) and, thus, might not be familiar with the advantages of telehealth, let alone its use—which also could be the case for the GPs [34].

Some considerations on self-reported data are appropriate. First, the questionnaires’ tendency towards, in particular, ceiling [21]—and, to a minor degree, floor—effects should be considered, especially in cases of interventions intending to increase the scores of shared decision-making. With the ceiling effect in six of nine items in a cross-sectional study, it is questionable whether the questionnaires’ responsiveness would allow for detecting expected increased mean scores [23]. On the other hand, authors of psychometric studies of the questionnaire argue that a narrow interpretation of results: *‘as ‘‘the higher, the better’’ is premature and short-sighted’* because the instrument only applies to clinical situations with more than one reasonable and/or evidence-based choice [19]. Hence, we must consider that a GP might feel obliged to decide what is best for a specific patient; the patient might interpret this as a lack of shared decision-making, whereas it could be a situation where shared decision-making is not applicable. Because the specific questionnaire only reflects the patients’ subjective views, the scores will only be high when the decisions made reflect the patients’ preferences [19]. Thus, a focus on information and the decision-making process is recommended rather than an instrumental focus on the outcome of a decision-making situation.

### 4.3. Strengths and limitations

As mentioned in the methods section, our study might include some challenges regarding the data collection, including self-selection and a skewed socio-economic representation. Moreover, the fact that the survey was conducted during Covid-19 with restricted access to GPs might have affected our result. Furthermore, the fact that the responses were based on heterogeneous decisions in a broadly defined general practice setting might also represent a weakness for our study’s rigorousness. However, our study sample is beyond the recommended ten respondents per item, contributing to a decrease in both the risk of case mismatch and datamining and, thus, increasing the generalizability of our results. Furthermore, we applied a validated and internationally acknowledged and often-used questionnaire, which also contributes to the study’s validity.

### 4.4. Implications for practice

Based on our results, we recommend that GPs—and perhaps other health professionals—pay specific attention to patients with COPD in decision-making processes. It is, of course, important for all patients to have a sense of being included in decisions regarding their own health and well-being, but it appears that patients with COPD are less involved. Moreover, being younger and female appears to contribute to being less involved in shared decision-making. We recommend that more attention is placed on the process rather than the outcome, and we suggest that shared decision-making is used as a specific tool.

## 5. Conclusion

Overall, patients are involved in shared decision-making in general practice; our results could, however, indicate a potential for more patient involvement in terms of shared decision-making in general practice. Minor nuances were found because patients with COPD were less involved in shared decision-making compared to patients with T2DM. Similarly, younger patients and women were less involved than older patients and men.

## References

[1] Chewning B, Bylund CL, Shah B, et al. Patient preferences for shared decisions: a systematic review. Patient Educ Couns. 2012;86(1):9–18. 21474265 10.1016/j.pec.2011.02.004 PMC4530615

[2] Kennedy AP, Rogers AE. Improving patient involvement in chronic disease management: the views of patients, GPs and specialists on a guidebook for ulcerative colitis. Patient Educ Couns. 2002;47(3):257–263. 12088604 10.1016/s0738-3991(01)00228-2

[3] Longtin Y, Sax H, Leape LL, et al. Patient participation: current knowledge and applicability to patient safety. Mayo Clin Proc. 2010;85(1):53–62. 20042562 10.4065/mcp.2009.0248 PMC2800278

[4] Florin J, Ehrenberg A, Ehnfors M. Patient participation in clinical decision-making in nursing: a comparative study of nurses' and patients' perceptions. J Clin Nurs. 2006;15(12):1498–1508. 17118072 10.1111/j.1365-2702.2005.01464.x

[5] Guadagnoli E, Ward P. Patient participation in decision-making. Soc Sci Med. 1998;47(3):329–339. 9681902 10.1016/s0277-9536(98)00059-8

[6] Wensing M, Grol R. Patients' views on healthcare: a driving force for improvement in disease. Management. Disease Management and Health Outcomes. 2000;7(3):117–125.

[7] Watanabe Y, Takahashi M, Kai I. Japanese cancer patient participation in and satisfaction with treatment-related decision-making: a qualitative study. BMC Public Health. 2008;8(1):77. 18302800 10.1186/1471-2458-8-77 PMC2291463

[8] Anderson C, Laubscher S, Burns R. Validation of the short form 36 (SF-36) health survey questionnaire among stroke patients. Stroke. 1996;27(10):1812–1816. 8841336 10.1161/01.str.27.10.1812

[9] Denis F, Lethrosne C, Pourel N, et al. Overall survival in patients with lung cancer using a web-application-guided follow-up compared to standard modalities: Results of phase III randomized trial. J Clin Oncol. 2016;34(18_suppl):LBA9006–LBA.

[10] Ernst J, Kuhnt S, Schwarzer A, et al. The desire for shared decision making among patients with solid and hematological cancer. Psycho-Oncology. 2011;20(2):186–193. 20238372 10.1002/pon.1723

[11] Health Do. Patient and public involvement in health: the evidence for policy implementation;. London: Department of Health; 2004.

[12] WHO;. Noncommunicable diseases country profiles 2014. 2014. July 2014.

[13] Schermer T, van Weel C, Barten F, et al. Prevention and management of chronic obstructive pulmonary disease (COPD) in primary care: position paper of the European forum for primary care. Qual Prim Care. 2008;16(5):363–377. 18973718

[14] Heltberg A, Andersen JS, Kragstrup J, et al. Social disparities in diabetes care: a general population study in Denmark. Scand J Prim Health Care. 2017;35(1):54–63. 28277046 10.1080/02813432.2017.1288702 PMC5361420

[15] Ahnfeldt-Mollerup P, Søndergaard J, Barwell F, et al. The relationships between use of quality-of-Care feedback reports on chronic diseases and medical engagement in general practice. Qual Manag Health Care. 2018;27(4):191–198. 30260925 10.1097/QMH.0000000000000188 PMC6166699

[16] REGIONERNES LØNNINGS- OG TAKSTNÆVN PRAKTISERENDE LÆGERS ORGANISATION. Overenskomst om almen praksis. [Collective agreement between Danish Regions and General Practice 2018, 2018.

[17] von Elm E, Altman DG, Egger M, STROBE Initiative, et al. The strengthening the reporting of observational studies in epidemiology (STROBE) statement: guidelines for reporting observational studies. Ann Intern Med. 2007;147(8):573–577. 17938396 10.7326/0003-4819-147-8-200710160-00010

[18] Simon D, Schorr G, Wirtz M, et al. Development and first validation of the shared decision-making questionnaire (SDM-Q). Patient Educ Couns. 2006;63(3):319–327. 16872793 10.1016/j.pec.2006.04.012

[19] Kriston L, Scholl I, Hölzel L, et al. The 9-item shared decision making questionnaire (SDM-Q-9). development and psychometric properties in a primary care sample. Patient Educ Couns. 2010;80(1):94–99. 19879711 10.1016/j.pec.2009.09.034

[20] Doherr H, Christalle E, Kriston L, et al. Use of the 9-item shared decision making questionnaire (SDM-Q-9 and SDM-Q-Doc) in intervention studies-A systematic review. PLoS One. 2017;12(3):e0173904–e. 28358864 10.1371/journal.pone.0173904 PMC5373562

[21] Hulbaek M, Jørgensen MJ, Mainz H, et al. Danish translation, cultural adaptation and validation of the shared decision making Questionnaire - Patient version (SDM-Q-9-Pat). EJPCH. 2018;6(3):438–446.

[22] Verhoeven V, Tsakitzidis G, Philips H, et al. Impact of the COVID-19 pandemic on the core functions of primary care: will the cure be worse than the disease? A qualitative interview study in flemish GPs. BMJ Open. 2020;10(6):e039674. 10.1136/bmjopen-2020-039674 PMC7306272 32554730

[23] de Vet HCW, Terwee CB, Mokkink LB, et al. Measurement in medicine: a practical guide. Cambridge: Cambridge University Press; 2011.

[24] Diendéré G, Farhat I, Witteman H, et al. Observer ratings of shared decision making do not match patient reports: an observational study in 5 family medicine Practices. Med Decis Making. 2021;41(1):51–59. 33371802 10.1177/0272989X20977885

[25] Scholl I, Kriston L, Dirmaier J, et al. Comparing the nine-item shared Decision-Making questionnaire to the OPTION scale - an attempt to establish convergent validity. Health Expect. 2015;18(1):137–150. 23176071 10.1111/hex.12022 PMC5060753

[26] Sundhedsstyrelsen. Rapport for specialet: Intern medicin: geriatri. [Report for the specialty: Internal Medicine: Geriatrics]. 2008.

[27] The General Data Protection Regulations (GDPR). Regulation (EU) 2016/679, 2016.

[28] Terwee CB, Bot SD, de Boer MR, et al. Quality criteria were proposed for measurement properties of health status questionnaires. J Clin Epidemiol. 2007;60(1):34–42. 17161752 10.1016/j.jclinepi.2006.03.012

[29] Early F, Lettis M, Winders SJ, et al. What matters to people with COPD: outputs from working together for change. NPJ Prim Care Respir Med. 2019;29(1):11. 30979889 10.1038/s41533-019-0124-z PMC6461642

[30] Schroedl CJ, Yount SE, Szmuilowicz E, et al. A qualitative study of unmet healthcare needs in chronic obstructive pulmonary disease. A potential role for specialist palliative care? Ann Am Thorac Soc. 2014;11(9):1433–1438. 25302521 10.1513/AnnalsATS.201404-155BC PMC4298987

[31] Halliwell J, Mulcahy P, Buetow S, et al. GP discussion of prognosis with patients with severe chronic obstructive pulmonary disease: a qualitative study. The British Journal of General Practice: The Journal of the Royal College of General Practitioners. 2004;54(509):904–908. 15588534 PMC1326107

[32] Berger BE, Kapella MC, Larson JL. The experience of stigma in chronic obstructive pulmonary disease. West J Nurs Res. 2011;33(7):916–932. 20940446 10.1177/0193945910384602 PMC6986355

[33] Laue J, Melbye H, Halvorsen PA, et al. How do general practitioners implement decision-making regarding COPD patients with exacerbations? An international focus group study. Int J Chron Obstruct Pulmon Dis. 2016;11:3109–3119. 27994450 10.2147/COPD.S118856 PMC5153277

[34] Abrams EM, Shaker M, Oppenheimer J, et al. The challenges and opportunities for shared decision making highlighted by COVID-19. J Allergy Clin Immunol Pract. 2020;8(8):2474–2480.e1. 32679348 10.1016/j.jaip.2020.07.003 PMC7358768

